# Delayed Chloroform Poisoning

**Published:** 1909-12-11

**Authors:** 


### ANÆSTHETICS.

### DELAYED CHLOROFORM POISONING.

WHEN a few years ago the attention of the profession was drawn by pathologists to the condition of lymphatism or status lymphaticus, as an antecedent of sudden deaths in children due to causes apparently trivial, it was especially in connection with anæsthetic fatalities that this curious diathesis (if that term may legitimately be applied to it) was described and attained notoriety. The result was that for a time almost every person dying under the influence of an anæsthetic was said to exhibit the status lymphaticus; and the natural result was the production of incredulity among some medical men as to the existence of such a state. Now that lymphatism is better understood, its characteristics are more clearly defined, and it is easy for an expert pathologist to decide in any given case whether the patient has or has not during life been the subject of the status lymphaticus, and whether, therefore, he was or was not liable to sudden death from a dose of anæsthetic which would not have been dangerous to a normal person.

In the same way researches of the last four years or so have taught us much about the condition known as delayed chloroform poisoning; and a similar danger has arisen as happened in the case of lymphatism. That is to say, a tendency is noticeable to ascribe every death within a few days of an operation to delayed chloroform poisoning, even when the very slenderest grounds for such a diagnosis are wanting. Surgeons are only human, and to be able to persuade oneself that responsibility for a catastrophe rests on someone else's shoulders is so human a failing that it is not surprising to hear of some such occurrences as one which lately took place in a certain hospital in London; a surgeon directed the erasure of a note in the registers which stated that "patient never rallied and died six hours after the operation," and the substitution of the statement that "patient died of delayed chloroform poisoning."

What then are the symptoms and signs by which the diagnosis of delayed chloroform poisoning may reasonably be conjectured? Briefly they are those of a condition of acidosis (see THE HOSPITAL, October 3, 1908, p. 15), or acetonæmia supervening from twelve hours to four days, most commonly between twenty-four and forty-eight hours, after the administration of chloroform, recovery from which has been apparently normal. Two types have been described; one resembling somewhat the acidosis of diabetic coma, the other more akin to acute yellow atrophy of the liver. The former begins with a stuporous condition of semi-coma, often ushered in by an attack of acute terror and wild delirium with screaming. Vomiting, which soon becomes of the so-called coffee-ground nature commences soon after; acetone is present in the urine, and can be smelt in the breath; and before long a profound coma sets in which ends, as a rule, in death. That is the clinical picture of a severe case, but there is little doubt that milder cases which do not end fatally are not very uncommon, though they are not so often reported. The other cases are characterised especially by jaundice and vomiting, with air hunger and distress which end in fatal coma. The urine contains bile pigments, and may contain acetone. In both types the post-mortem appearances show fatty degeneration of the most important viscera, the liver, kidneys, heart, etc. The icteric type is most frequently seen in adults, the comatose type in infants.

Now temporary acetonuria follows the administration of both chloroform and ether very frequently; in fact, quite half the number of anæsthetised patients display the nitro-prusside reaction in their urine for a short period. But only a very minute proportion indeed get delayed chloroform poisoning; and it is particularly to be remarked that of those who do suffer from this remarkable syndrome a certain number do not show acetonuria at all. Quite recently Dr. T. C. Somerville * reports three fatal cases under observation at Huddersfield, in two of which no acetone was detected. These cases were so typical that the descriptions given of them might almost serve for a text-book summary of the condition. In the acetonuric patient, a girl of four years old, an osteotomy of the femur for genu valgum was done under A.C.E., of which but six drachms were administered. Thirteen hours after the conclusion of the operation there was a single act of vomiting, after which the child fell asleep. Four hours later there occurred more vomiting of "coffee-ground" material, with a fast and weak pulse, and the gradual onset of coma; the state of dulness alternating with apathy is said to describe the mental condition. Twenty hours later the temperature had risen to 106° F., the pulse was uncountable, and respiration was very rapid: there was slight abdominal distension, and some tenderness. Persistent vomiting proceeded steadily until death took place: no treatment produced the smallest effect.

In diabetics, and in the subjects of severe and prolonged malnutrition, delayed chloroform poisoning is said to be particularly feared. Diseases of the liver are also held to predispose to acidosis, and it has also been held that a previous anæsthesia (with chloroform) makes a second one more likely to set up this disorder. It has been suggested by Hunter that the explanation of acidosis, whether as a result of chloroform or of other causes, is the formation of oxybutyric, diacetic, and other fatty acids from the decomposition of fat in the liver: and this decomposition is held to be rendered necessary by the failure of the hepatic cells when poisoned by chloroform properly to deal with proteids. It has consequently been supposed that first glycogen and then fats are burnt up in the liver to supply the energy required for the body; and on

* *Lancet*, July 10, 1909, p. 81.

this hypothesis means have been devised for increasing the glycogen content of the liver preliminary to the administration of an anæsthetic. Dr. Beddard accordingly advised the effect of feeding patients on dextrose, and Messrs. Wallace and Gillespie† found this measure of prophylaxis more effective in preventing vomiting and acetonuria after chloroform or ether than the suggestion of Beasley‡ that sodium bicarbonate should be used. It may be added here that delayed poisoning similar in every respect to that seen after chloroform inhalation has very occasionally been known after ether, ethyl chloride, and even nitrous oxide gas.

Another theory to explain these cases is based on the assumption, not of a special vulnerability of the hepatic tissue of the patient to chloroform, but of a pre-existing fatty condition of the liver before the anæsthetic is given. In support of this Mr. H. T. Ashby§ remarks that ether does not cause any fatty changes in the liver, yet may be followed by effects similar to those of delayed chloroform poisoning; that it is difficult to believe that the extreme fatty changes seen post-mortem can be produced in 48 hours or even less; and that in cyclic vomiting in children, a condition closely allied to delayed chloroform poisoning, the liver is known to be very fatty. Against it he points out that numbers of patients with fatty livers, and many children who suffer from cyclical vomiting, are anæsthetised without any dangerous after effects.

Finally, with regard to treatment, the attempt at prophylaxis by means of half an ounce of glucose given four hourly for six doses before operation seems worth a trial: it is simple, inexpensive, and not likely to be irksome to the patient. When intractable vomiting has once set in subcutaneous saline infusion is one of the best ways of stimulating the patient: or intravenous infusion with a solution of bicarbonate of soda, or washing out the stomach with the same drug may be tried. For less severe cases champagne, brandy, and iced food may be ordered; and a smart purge is in all cases advisable if it can be retained. Beddard suggested intravenous transfusion with dextrose; but other authors seem to think that while dextrose is the best prophylactic, sodium bicarbonate is of more value when acidosis has actually arisen. It must be remembered that the blood never loses its alkaline reaction completely: the condition of acidosis is one, not of acidity of the blood, but simply of lessened alkalinity.

† *Lancet*, December 5, 1908, p. 1665.
‡ *British Medical Journal.*
§ *Medical Chronicle*, November 1908, p. 81.

